# From lipoprotein metabolism to blood clotting: Highlights of the 2025 Fredrickson lipid research conference

**DOI:** 10.1016/j.jlr.2026.101041

**Published:** 2026-04-16

**Authors:** Alexis N. Smith, Mirza A. Beg, Woosuk Steve Hur, David Ginsburg, Iftikhar J. Kullo, Sean S. Davies, Ira Tabas, Jesse W. Williams, Chieko Mineo, Bishuang Cai, Rana K. Gupta, Jaume Amengual, Anna Schwendeman, Gregory A. Graf, Mary Sorci Thomas, Ze Zheng

**Affiliations:** 1 Department of Thrombosis and Hemostasis, Versiti Blood Research Institute, Milwaukee, WI, USA; 2 Cardiovascular Research Center, Medical College of Wisconsin, Milwaukee, WI, USA; 3 Department of Medicine, Medical College of Wisconsin, Milwaukee, WI, USA; 4 Department of Pathology and Laboratory Medicine, University of North Carolina at Chapel Hill, Chapel Hill, NC, USA; 5 Lineberger Comprehensive Cancer Center, University of North Carolina at Chapel Hill, Chapel Hill, NC, USA; 6 UNC Blood Research Center, University of North Carolina at Chapel Hill, Chapel Hill, NC, USA; 7 Life Sciences Institute, University of Michigan, Ann Arbor, MI, USA; 8 Department of Internal Medicine, University of Michigan, Ann Arbor, MI, USA; 9 Department of Human Genetics, University of Michigan, Ann Arbor, MI, USA; 10 Department of Pediatrics, University of Michigan, Ann Arbor, MI, USA; 11 Department of Cardiovascular Medicine, Mayo Clinic, Rochester, MN, USA; 12 Division of Clinical Pharmacology, Vanderbilt Institute of Chemical Biology, Vanderbilt University, Nashville, TN, USA; 13 Department of Medicine, Columbia University Irving Medical Center, New York, NY, USA; 14 Department of Pathology and Cell Biology, Columbia University Irving Medical Center, New York, NY, USA; 15 Department of Physiology and Cellular Biophysics, Columbia University Irving Medical Center, New York, NY, USA; 16 Center for Immunology, Department of Integrative Biology & Physiology, University of Minnesota Medical School, Minneapolis, MN, USA; 17 Department of Pediatrics and Cell Biology, Center for Pulmonary and Vascular Biology, University of Texas Southwestern Medical Center, Dallas, TX, USA; 18 Department of Medicine, University of California at Los Angeles, Los Angeles, CA, USA; 19 Division of Endocrinology, Department of Medicine, Duke Molecular Physiology Institute, Duke University, Durham, NC, USA; 20 Division of Nutritional Sciences, University of Illinois Urbana-Champaign, Champaign, IL, USA; 21 Department of Food Science and Human Nutrition, University of Illinois Urbana-Champaign, Champaign, IL, USA; 22 Department of Pharmaceutical Sciences, University of Michigan, Ann Arbor, MI, USA; 23 Saha Cardiovascular Research Center, College of Medicine, University of Kentucky, Lexington, KY, USA; 24 Department of Physiology, College of Medicine, University of Kentucky, Lexington, KY, USA; 25 Barnstable Brown Diabetes Center, UK Healthcare, Lexington, KY, USA; 26 Department of Pharmacology and Toxicology, Medical College of Wisconsin, Milwaukee, WI, USA; 27 Department of Physiology, Medical College of Wisconsin, Milwaukee, WI, USA

**Keywords:** adipose tissue, biomarkers, cardiovascular diseases, efferocytosis, inflammation, lipids, lipoproteins

## Abstract

The biannual Fredrickson Lipid Research conference took place in person in Milwaukee, Wisconsin, from September 3-5, 2025. Each conference highlights the most advanced basic and translational research in lipids and lipoprotein metabolism. As with each Fredrickson Lipid Research conference, the overall theme is focused on “Lipid Metabolism, Lipoproteins, and Atherosclerosis,” and this year included a special scientific session exploring the connections between Blood Clotting and Lipid Metabolism. This session underscored ongoing research aimed at explaining the mechanisms by which dyslipidemia alters the coagulation system and drives thrombotic cardiovascular disease. Other scientific session themes at the conference included Biomarkers in Cardiovascular Diseases, Efferocytosis and Inflammation, Regulation of Lipoproteins, Adipocyte Plasticity in Cardiometabolic Diseases, and Novel Therapeutic Strategies for Atherosclerosis. From the six moderated scientific sessions, invited speakers provided summaries of their work to showcase their contributions to the lipid and lipoprotein metabolism fields. This review article aims to serve as a resource for readers to learn more about ongoing research in lipid and lipoprotein metabolism and to encourage future studies and collaborations that advance the field.

The 2025 Fredrickson Lipid Research Conference took place in person in Milwaukee, WI, from September 3-5, 2025, hosted by the Medical College of Wisconsin and Versiti Blood Research Institute. Historically, this conference evolved from the Southeast Lipid Research Conference, which started in 1992 and was then renamed in 2019 to honor the work of Dr. Donald S. Fredrickson in lipids and lipoprotein metabolism. Dr. Fredrickson was a renowned lipid specialist who made significant contributions to the field, such as the Fredrickson classification of lipid disorders, and served as the 11^th^ director of the National Institutes of Health (NIH) from 1975 to 1981, where he continued to impact and push forward innovative science. The conference honors the legacy of Dr. Fredrickson and is held biannually, highlighting new advances in research in the lipid and lipoprotein metabolism fields.

Each Fredrickson Lipid Research conference has a unique focus on highlighting the most advanced basic and translational research in lipids and lipoprotein metabolism. This year, the overall theme of the conference was “Lipid Metabolism, Lipoproteins, and Atherosclerosis.” We also offered a special scientific session looking into the connections between Blood Clotting and Lipid Metabolism. This session was special because it highlighted the connections between dyslipidemia, and the effects on the blood clotting system in relation to thrombotic cardiovascular diseases. Dysregulated lipid metabolism can influence both clot formation and clot lysis, increasing the risk of thrombotic events. Research presented at the conference demonstrated that deficiencies in certain blood-clotting factors can alter lipoprotein metabolism. Together, these findings highlight why the interplay between blood-clotting factors and lipid metabolism emerged as a central theme of the conference and underscore the need for continued investigation into their roles in thrombotic cardiovascular disease. Other scientific session themes at the conference included Biomarkers in Cardiovascular Diseases, Efferocytosis and Inflammation, Regulation of Lipoproteins, Adipocyte Plasticity in Cardiometabolic Diseases, and Novel Therapeutic Strategies for Atherosclerosis. This review article provides a summary of the work in these scientific sessions presented by invited speakers to highlight major scientific advancements in the field.

## Blood Clotting and Lipid Metabolism

### Plasminogen activation system in the pathogenesis of obesity and metabolic syndrome

#### Presented by Dr. Woosuk Steve Hur

**Fig. 1:**
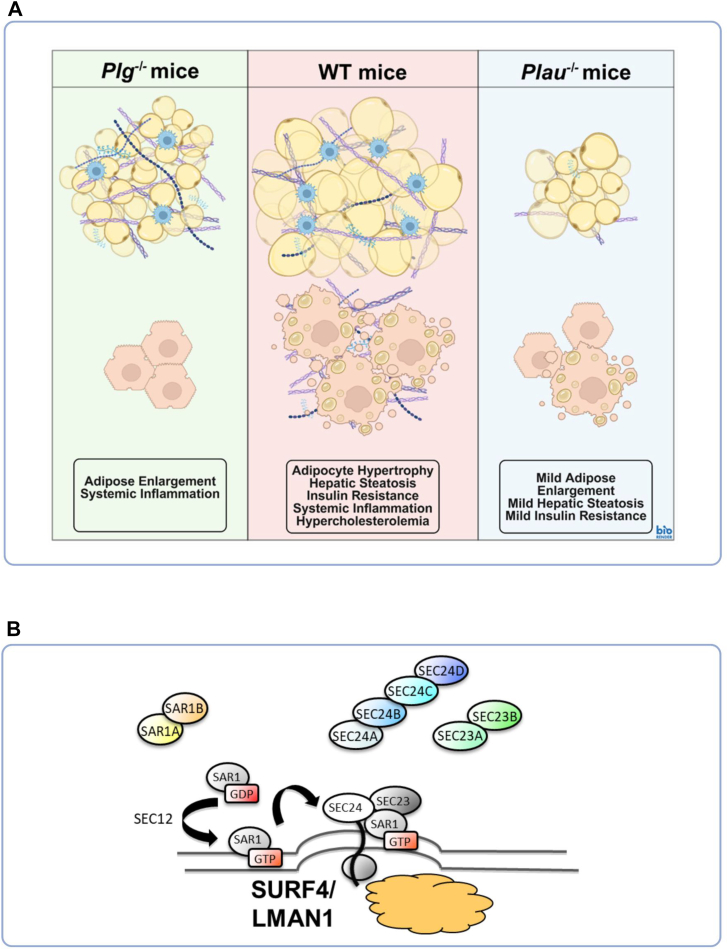
Blood Clotting and Lipid Metabolism. A: Dr. Woosuk Steve Hur’s work at the University of North Carolina at Chapel Hill examined the role of the fibrinolytic system in obesity-associated metabolic dysfunction. In high-fat diet models, deficiency of fibrinolytic components altered metabolic outcomes: plasminogen-deficient mice (Plg^−/−^) are protected from hepatic steatosis, insulin resistance, and hypercholesterolemia, while urokinase plasminogen activator-deficient mice (Plau^−/−^) have attenuated diet-induced weight gain and metabolic dysfunction in comparison to wild-type (WT) mice. B: Dr. David Ginsberg’s work at the University of Michigan identified LMAN1 (Lectin, mannose binding 1) and MCFD2 (Multiple coagulation factor deficiency protein 2), referred to as the LMAN1/MCFD2 complex, as the cargo receptor for the coagulation factors Factor V (F5) and Factor VIII (F8). The cargo receptor Surfeit locus protein 4 (SURF) mediates Proprotein convertase subtilisin/kexin type 9 (PCSK9) secretion, and mice deficient in the Coat protein complex II (COPII) component of SEC24 homolog A (SEC24 A) show reduced PCSK9 secretion and lower cholesterol levels.

Obesity is strongly and causally linked to an elevated incidence and severity of cardiovascular disease, the leading cause of disability and death worldwide (1). Obesity is associated with significantly increased coagulation activation and reduced fibrinolytic activity (1, 2, 3). Recent studies indicate that there is a reciprocal role of fibrinolytic system components modulating the development of high-fat diet (HFD)-induced weight gain and associated metabolic pathologies such as metabolic dysfunction-associated steatotic liver disease, type 2 diabetes, and hypercholesterolemia (4, 5). In mouse models of diet-induced obesity, wild-type (WT) mice develop adipocyte hypertrophy, hepatic steatosis, insulin resistance, and hypercholesterolemia, while mice deficient in plasminogen (*Plg*^*−/−*^ mice) have suppressed HFD-induced hepatic steatosis, insulin resistance, and hypercholesterolemia (Fig. 1A). Furthermore, mice deficient in urokinase plasminogen activator (*Plau*^*−/−*^ mice) have attenuated HFD-induced development of diet-induced weight gain and associated metabolic dysfunction.

### Cargo receptors in the ER: from clotting factors to cholesterol regulation

#### Presented by Dr. David Ginsburg

Genetics of the rare bleeding disorder, combined deficiency of Factor V (F5) and Factor VIII (F8), identified LMAN1 (Lectin, mannose binding 1), and MCFD2 (Multiple coagulation factor deficiency protein 2), referred to as the LMAN1/MCFD2 complex, as their cargo receptor (mediating ER/Golgi transport) (6, 7). The LMAN1/MCFD2 complex has a limited number of other cargoes (8). Mice deficient in the Coat protein complex II (COPII) component SEC24 homolog A (SEC24A) have low cholesterol, resulting from reduced Proprotein convertase subtilisin/kexin type 9 (PCSK9) secretion, the latter mediated by the cargo receptor, Surfeit locus protein 4 (SURF4) (Fig. 1B) (6, 7). SURF4 mediates the ER/Golgi transport of multiple other cargoes, including apolipoprotein B (8).

## Biomarkers in Cardiovascular Diseases

### Investigating biomarkers of cardiovascular risk

#### Presented by Dr. Iftikhar J. Kullo

Inflammation and dyslipidemia are the two major pathways of atherogenesis. These influence plaque formation and evolution with time. Early atheroma may progress to a stable fibrous plaque or into a thin cap fibro atheroma, also known as vulnerable plaque. Generally, plaque burden increases with age, but the slope of the increase may vary in an individual depending on exposure to risk factors as well as genetic background (9). There is great interest in measuring plaque quantity, quality, and behavior to better predict the risk of adverse cardiovascular events in an individual.

**Fig. 2:**
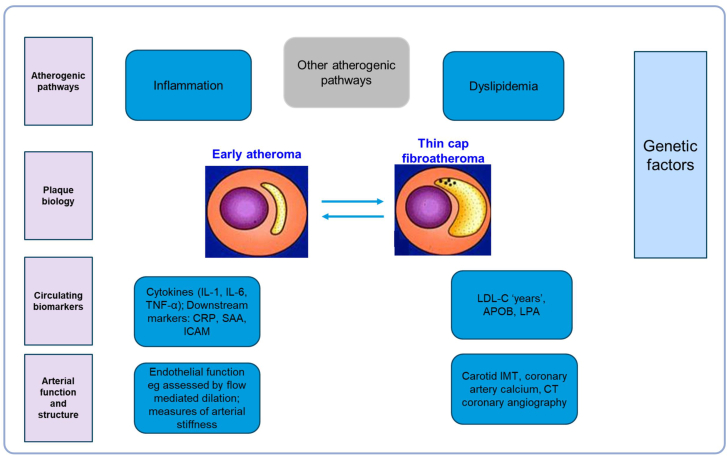
Biomarkers in cardiovascular diseases. Dr. Iftikhar J. Kullo’s work at the Mayo Clinic described the pathways of atherogenesis. Inflammation and dyslipidemia drive plaque formation and evolution, from early atheroma to stable or vulnerable plaques. Circulating inflammatory markers (e.g., IL-1, IL-6, and TNF-α) and lipid markers (e.g., LDL cholesterol, LDL-C, and Apolipoprotein B, apoB) reflect systemic risk. In addition, genetic predisposition, arterial function, and arterial structure collectively influence cardiovascular risk, highlighting the need for an integrated assessment to assess cardiovascular risk in individuals.

Circulating markers of inflammation include cytokines such as interleukin-1 and -6 and TNF-alpha, as well as downstream markers such as C-reactive protein, Serum Amyloid A, and adhesion molecules (Fig. 2) (10). Lipid markers, beyond the traditional lipid profile, include assessing cumulative exposure to LDL cholesterol, Apolipoprotein B, and lipoprotein (a) (11, 12). Genetic predisposition can be assessed by polygenic risk scores as well as by testing for monogenic etiology when appropriate, such as for familial hypercholesterolemia. The final common pathway of atherogenic factors is to alter arterial function and structure. Arterial function can be assessed by measures of endothelial function, such as flow-mediated dilatation, as well as aortic stiffness. Arterial structure can be assessed by carotid intima-media thickness, coronary calcification, and by CT angiography. In summary, the complexity of plaque formation and evolution with time means that multiple inputs are needed for refining cardiovascular risk in an individual, including circulating biomarkers, polygenic risk scores, *and measurements of arterial function and structure.*

## Efferocytosis and Inflammation

### The role of NAPE-PLD in the regulation of macrophage function and atherosclerosis

#### Presented by Dr. Sean Davies

Efferocytosis, the phagocytic clearance of apoptotic cells, reprograms macrophages to promote tissue resolution. Impaired efferocytosis is a hallmark of atherosclerotic lesions and leads to increased necrotic core area and thinning of the fibrous cap. The mechanisms driving impaired efferocytosis during atherosclerosis remain to be fully elucidated.

**Fig. 3:**
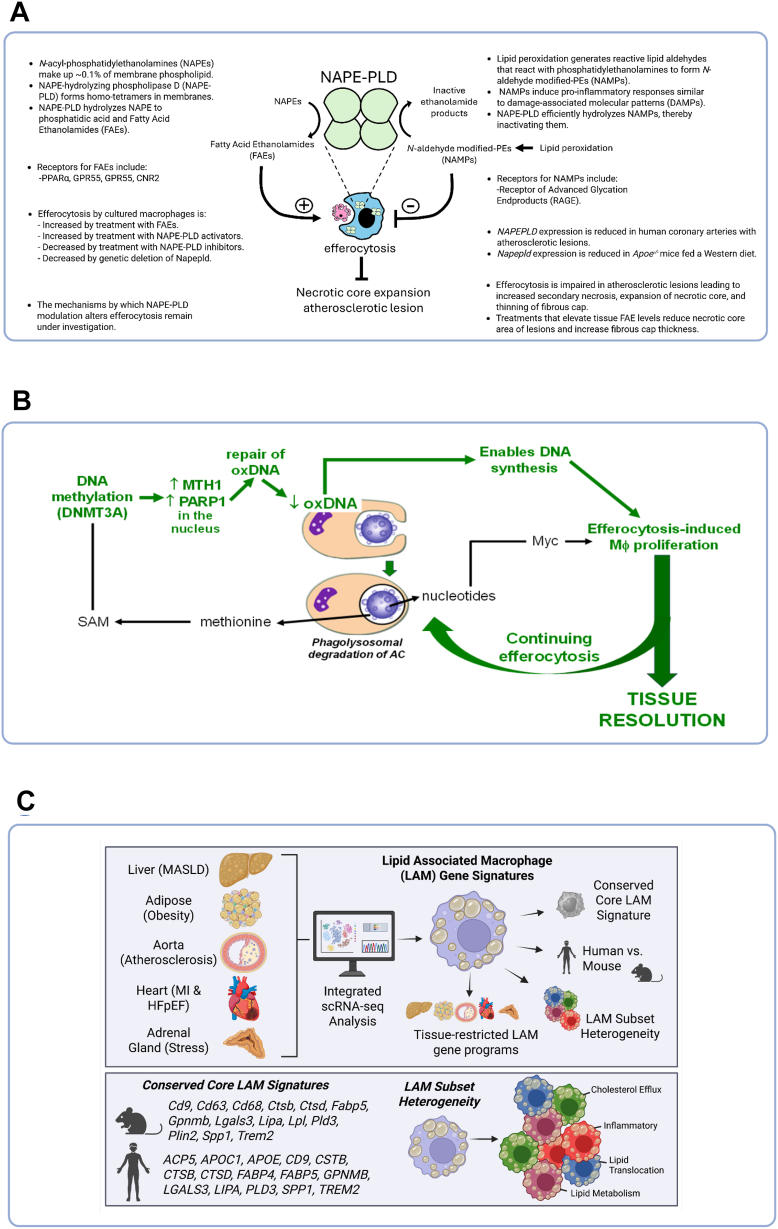
Efferocytosis and inflammation. A: Dr. Sean Davies’s work at Vanderbilt University examined the role of *N*-acyl-phosphatidylethanolamine-hydrolyzing phospholipase D (NAPE-PLD) in macrophage efferocytosis and plaque stability. Impaired efferocytosis in atherosclerotic lesions increases necrotic core size and thins the fibrous cap. NAPE-PLD expression is reduced in atherosclerotic lesions. Treatment with FAEs and NAPE-PLD activators increased efferocytosis, promoting plaque resolution and fibrous cap stability, while NAPE-PLD inhibitors or genetic deletion decreased efferocytosis. B: Dr. Ira Tabas’s work at Columbia University examined the pathway of efferocytosis-mediated oxidized DNA repair and macrophage proliferation. Efferocytosis lowered oxidized DNA in macrophages via DNMT3A (DNA methyltransferase 3 alpha) and the repair enzymes PARP1 (Poly (ADP-ribose) polymerase 1) and MTH1 (MuT Homolog 1). This pathway drove efferocytosis-induced macrophage proliferation (EIMP), expanded the pool of pro-resolving macrophages, and supported tissue repair for atherosclerotic lesion regression. C: Dr. Jesse W. Williams’s work at the University of Minnesota described the transcriptional profiling of lipid-associated macrophages (LAMs). Comparative scRNA-seq analysis of LAMs across mice and human tissues identified a conserved core LAM gene signature, four transcriptionally distinct LAM subpopulations, and tissue-specific gene programs, providing a resource for understanding LAM heterogeneity and therapeutic targeting in chronic inflammation.

One potential mechanism is reduced *N*-acyl-phosphatidylethanolamine-hydrolyzing phospholipase D (NAPE-PLD) activity, as human arteries with atherosclerotic lesions show reduced NAPE-PLD expression (Fig. 3A) (13). NAPE-PLD generates Fatty Acid Ethanolamides (FAEs) and degrades *N*-aldehyde-modified-phosphatidylethanolamines (NAMPs) (14). FAEs and NAPE-PLD activators increased efferocytosis by cultured macrophages, while NAPE-PLD inhibitors or genetic deletion inhibited efferocytosis (15). Raising FAE levels in atherosclerotic mice reduced the necrotic core area and increased the fibrous cap thickness (16). The mechanisms by which NAPE-PLD modulation alters macrophage efferocytosis remain under active investigation.

### DNA repair links two cargo pathways in the efferocytosis-resolution cycle

#### Presented by Dr. Ira Tabas

Efferocytosis lowers oxidized DNA in macrophages through a process requiring DNMT3A (DNA methyltransferase 3 alpha) and two oxidized DNA repair enzymes, PARP1 (Poly(ADP-ribose) polymerase 1) and MTH1 (MuT Homolog 1) (Fig. 3B) (17, 18). This pathway is required for a key resolution process called efferocytosis-induced macrophage proliferation (EIMP), which expands the pool of pro-resolving macrophages and is critical for tissue resolution in vivo (17). In atherosclerosis regression, loss of function of macrophage DNMT3A caused higher 8-OHdG (8-Hydroxy-2′-deoxyguanosine) and lower nuclear PARP1 and MTH1 in lesional macrophages, impaired lesional EIMP, and thinner fibrous caps. In summary, efferocytosing macrophages possess a unique oxidized DNA repair pathway that enables pro-resolving macrophage proliferation and tissue repair, with relevance to atherosclerosis regression.

### Lipid-associated macrophage (LAM) transcriptional Programming and heterogeneity

#### Presented by Dr. Jesse W. Williams

Lipid-associated macrophages (LAMs) are key regulators of chronic inflammation (18) and an emerging therapeutic target (19, 20). Dr. William’s laboratory performed comparative transcriptional analysis of LAMs isolated from organs across chronic inflammatory settings in both mouse and human (Fig. 3C) (21). Analysis of merged scRNA-seq LAM datasets identified a conserved core LAM gene signature, which was largely shared between mouse and human LAMs. In addition, four LAM subpopulations were transcriptionally defined by the enrichment of unique pathways, and LAM heterogeneity with tissue-restricted gene expression programs was identified. Collectively, these data represent a key resource for the analysis and therapeutic targeting of LAMs in chronic inflammatory settings.

## Regulation of Lipoproteins

### SR-BI in cardiometabolic health and disease

#### Presented by Dr. Chieko Mineo

**Fig. 4:**
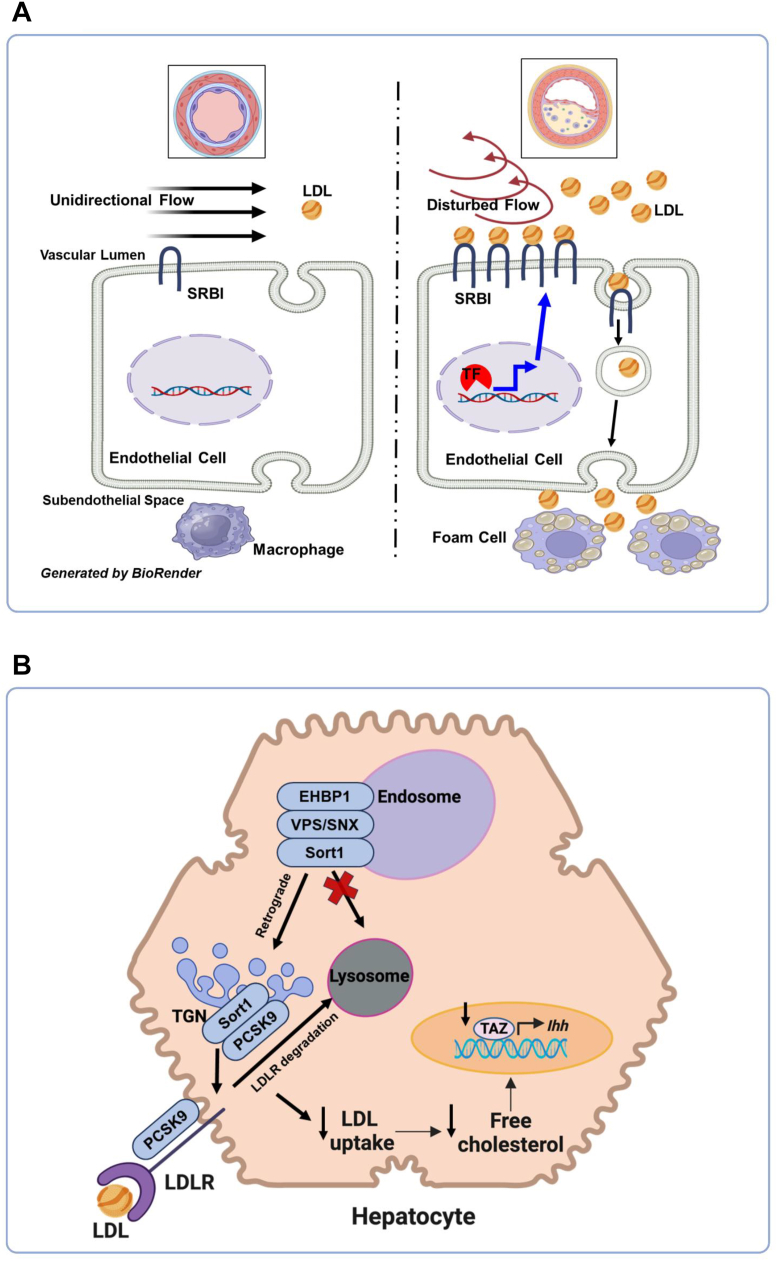
Regulation of Lipoproteins. A: Dr. Chieko Mineo’s work at the University of Texas demonstrated that endothelial Scavenger Receptor Class B Type I (SR-BI) played a role in low-density lipoprotein (LDL) transport and atherogenesis. In areas of the endothelial layer that were exposed to disturbed flow and hyperlipidemia, transcription factors such as Hypoxia inducible factor 1 subunit alpha (HIF1α) upregulated SR-BI, promoting subendothelial LDL accumulation and foam cell formation, contributing to atherosclerosis. B: Dr. Bishuang Cai’s work at the University of California, Los Angeles, examined how the EH domain binding protein 1 (EHBP1) forms a complex with retromer and Sortilin (Sort1) and regulates PCSK9 secretion and cholesterol homeostasis. Secreted PCSK9 targeted the low-density lipoprotein receptor (LDLR) for lysosomal degradation, resulting in reduced LDL uptake, decreased hepatic cholesterol levels, and lower Transcriptional Coactivator with PDZ-binding motif (TAZ), which is beneficial for liver homeostasis.

Prior work from Dr. Mineo’s laboratory and others demonstrated that Scavenger Receptor Class B Type I (SR-BI) mediates the transport of low-density lipoproteins (LDL) across endothelial cells (22, 23). A loss of endothelial SR-BI results in attenuation of atherosclerosis in hyperlipidemic mice (22). Single-cell transcriptomic analysis of human coronary arteries found that SR-BI expression in endothelial cells is transcriptionally upregulated by hyperlipidemia and disturbed flow (Fig. 4A). In the area where the endothelial layer was exposed to disturbed flow and high levels of LDL (right side of the schematic), transcription factors (TF), such as Hypoxia inducible factor 1 subunit alpha (HIF1α), upregulated SR-BI expression, which in turn promoted LDL build-up in the subendothelial space, likely contributing to foam cell formation and atherosclerosis.

### Membrane trafficking in cholesterol

#### Presented by Dr. Bishuang Cai

Genome-wide association studies (GWAS) indicate that EH domain binding protein 1 (EHBP1) is associated with LDL cholesterol (24, 25). Mechanistically, EHBP1 forms a complex with retromer and Sortilin 1 (Sort1) on endosomes and facilitates retromer-mediated retrograde transport of Sort1 to the trans-Golgi network (TGN), where Sort1 promotes PCSK9 secretion (Fig. 4B) (26). Secreted PCSK9 targeted the low-density lipoprotein receptor (LDLR) for lysosomal degradation, resulting in reduced LDL uptake, decreased hepatic cholesterol levels, and lower Transcriptional Coactivator with PDZ-binding motif (TAZ), which is beneficial for liver homeostasis (26).

## Adipocyte Plasticity in Cardiometabolic Disease

### Adipose tissue expansion and remodeling in cardiometabolic disease

#### Presented by Dr. Rana Gupta

Nearly half of adults and one-third of children worldwide will be overweight or obese by 2050 (27), placing hundreds of millions at risk for developing cardiometabolic disorders. Yet a significant portion of individuals with obesity will maintain cardiometabolic health, at least for a longer duration (28).

**Fig. 5:**
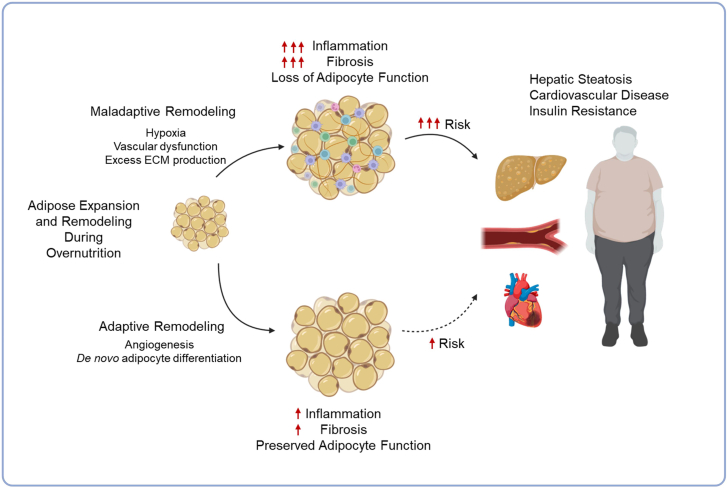
Adipocyte plasticity in cardiometabolic disease. Dr. Rana Gupta’s work at Duke University described white adipose tissue remodeling in metabolic health and disease. In metabolically healthy obesity (MHO), adipose tissue remodeling retained adaptive remodeling, including angiogenesis and adipogenesis, supporting functional adipocytes and energy storage. In contrast, in metabolically unhealthy obesity (MUO), loss of adipose plasticity led to fibrosis, inflammation, adipocyte dysfunction, and systemic metabolic decline, highlighting the role of WAT remodeling over mass in driving disease.

Comparisons of metabolically healthy obesity (MHO) to metabolically unhealthy obesity (MUO) pointed to white adipose tissue (WAT) remodeling, rather than mass, as a driver of metabolic decline (Fig. 5). Healthy adaptive adipose remodeling includes angiogenesis and de novo adipocyte differentiation (“adipogenesis”) (29). These events support the growth and energy-storing capacity of the tissue, ensuring retention of functional adipocytes (30). Loss of this plasticity leads to maladaptive remodeling of expanding adipose tissue, characterized by fibrosis, inflammation, and adipocyte dysfunction. Loss of protective adipokines, ectopic lipid deposition, and systemic inflammation can contribute to the development of disease. Ongoing efforts focus on understanding the physiological and environmental determinants of adipose plasticity. A deeper understanding of the interindividual differences in metabolic outcome in obesity can yield a mechanistic foundation for precision medicine and inform strategies to promote metabolic resilience throughout the lifespan.

## Novel Therapeutic Strategies for Atherosclerosis

### Role of carotenoids and vitamin A in atherosclerosis

#### Presented by Dr. Jaume Amengual

**Fig. 6:**
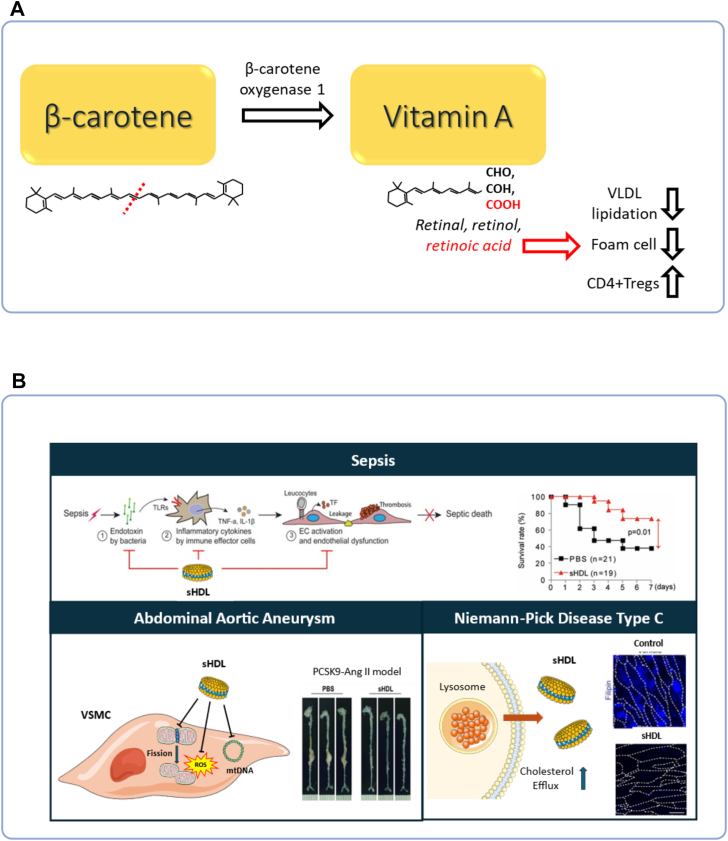
Novel therapeutic strategies for atherosclerosis. A: Dr. Jaume Amengual’s work at the University of Illinois investigated β-Carotene metabolism and cardiovascular protection. Cleavage of β-carotene by β-carotene oxygenase 1 produces vitamin A, whose active form, retinoic acid, promotes lipid oxidation and suppresses inflammation. β-Carotene activity and supplementation lowered cholesterol, reduced hepatic lipoprotein lipidation, limited foam cell formation, and enhanced differentiation of regulatory T cells for atherosclerosis resolution. B: Dr. Anna Schwendeman’s work at the University of Michigan outlined the therapeutic applications of synthetic HDL (sHDL) nanoparticles. Beyond promoting cholesterol efflux, sHDL protected against multiple diseases: in sepsis, it neutralized endotoxin, limited inflammation, preserved endothelial function, and improved survival; in abdominal aortic aneurysm (AAA), it enhanced VSMC mitochondrial function and reduced AAA incidence; in Niemann-Pick disease, it cleared cellular cholesterol and improved liver function.

The cleavage of β-carotene to form vitamin A is the first and limiting step in the production of vitamin A in animals. Retinoic acid, the transcriptionally active form of vitamin A, stimulates lipid oxidation and mitigates pro-inflammatory signals. In the context of atherosclerosis and cardiovascular health, β-carotene oxygenase 1 activity is associated with lower cholesterol in people (31) and reduced hepatic lipoprotein lipidation (Fig. 6A) (32). β-carotene supplementation also delayed atherogenesis (32) and accelerated atherosclerosis resolution (33) by mitigating foam cell formation and promoting the differentiation of regulatory T cells (Tregs), respectively.

### Beyond atherosclerosis: Broaden therapeutic applications of synthetic high-density lipoprotein

#### Presented by Dr. Anna Schwendeman

Traditionally, synthetic high-density lipoprotein (sHDL) nanoparticles have been developed to treat atherosclerotic diseases, focusing on their cholesterol-efflux functions. By harnessing broader protective functions of HDL, Dr. Schwendeman’s group has extended the applications of sHDL therapy to multiple disease areas. In the sepsis model, sHDL nanoparticles were found to effectively neutralize endotoxin, reduce inflammatory responses, and protect endothelial function, leading to increased survival rate in a cecal ligation and puncture murine model of sepsis (Fig. 6B) (34). In abdominal aortic aneurysm (AAA), administration of sHDL improved mitochondrial function of vascular smooth muscle cells (VSCM) and decreased AAA incidence (35). Additionally, sHDL treatment effectively removed accumulated cholesterol from Niemann-Pick C fibroblasts in vitro and improved liver function in vivo, suggesting the therapeutic potential of sHDL in Niemann-Pick diseases (36).

## Summary

Invited speakers from the six moderated scientific sessions prepared summaries of their work to highlight their contributions to the lipid and lipoprotein metabolism fields. Each session highlighted advancements being made in the fields of lipid metabolism, lipoproteins, and atherosclerosis. Overall, the conference emphasized blood clotting and lipid metabolism, highlighted by the presentations, which demonstrated that deficiencies in certain blood-clotting factors can alter lipoprotein metabolism. In addition, several presentations focused on efferocytosis and inflammation, providing new insights into how macrophage function is affected in atherosclerotic cardiovascular disease. Finally, the meeting identified key areas for future work, including expanding the biomarkers for measuring cardiovascular risk, clarifying mechanisms that regulate lipoproteins, and advancing understanding of adipocyte plasticity. Together, these take-home messages reflect a rapidly evolving field and point toward new opportunities for advancing research in lipid and lipoprotein metabolism.

We invite readers to draw on this new knowledge from this resource, allowing them to further their own research or establish new research directions. The goal of this educational resource aligns with the purpose of the conference, which is to disseminate new knowledge in the field and connect researchers together to further advance research in lipid and lipoprotein metabolism.

## Conflict of Interest

The authors declare that they do not have any conflicts of interest with the content of this article.
